# Evolving SAXS versatility: solution X-ray scattering for macromolecular architecture, functional landscapes, and integrative structural biology

**DOI:** 10.1016/j.sbi.2019.04.004

**Published:** 2019-06-13

**Authors:** Chris A Brosey, John A Tainer

**Affiliations:** 1 Molecular and Cellular Oncology and Cancer Biology, The University of Texas M. D. Anderson Cancer Center, Houston, TX 77030, USA; 2 MBIB Division, Lawrence Berkeley National Laboratory, Berkeley, CA 94720, USA

## Abstract

Small-angle X-ray scattering (SAXS) has emerged as an enabling integrative technique for comprehensive analyses of macromolecular structures and interactions in solution. Over the past two decades, SAXS has become a mainstay of the structural biologist’s toolbox, supplying multiplexed measurements of molecular shape and dynamics that unveil biological function. Here, we discuss evolving SAXS theory, methods, and applications that extend the field of small-angle scattering beyond simple shape characterization. SAXS, coupled with size-exclusion chromatography (SEC-SAXS) and time-resolved (TR-SAXS) methods, is now providing high-resolution insight into macromolecular flexibility and ensembles, delineating biophysical landscapes, and facilitating high-throughput library screening to assess macromolecular properties and to create opportunities for drug discovery. Looking forward, we consider SAXS in the integrative era of hybrid structural biology methods, its potential for illuminating cellular supramolecular and mesoscale structures, and its capacity to complement high-throughput bioinformatics sequencing data. As advances in the field continue, we look forward to proliferating uses of SAXS based upon its abilities to robustly produce mechanistic insights for biology and medicine.

## Introduction

Structural biology has long interpreted the language of cell biology by illuminating dynamic molecular architectures, revealing how structure encodes biological function and is shaped by genetic sequence and the fundamental physical chemistry underlying evolved molecular mechanisms. The advent of the ‘omics’ era of biology has significantly expanded the landscape for linking sequence to complex cellular phenotypes via macromolecular shapes, assemblies, and dynamics. Efficient methods to delineate molecular conformations regulating interactions and chemistry in near physiological environments are thus paramount in this new era of molecular and cellular biology. Following its renaissance over the past two decades, the field of biological small-angle X-ray scattering (SAXS) continues to illuminate biomolecular assemblies and their biophysical states with information-rich experiments, yielding key mechanistic insights into macromolecular functions of cellular machinery. The expansion of dedicated biological SAXS beamlines [1–6], greater use of SAXS combined with crystallography [7^••^], standardization of publication guidelines for X-ray scattering data [8^•^,^9^,^10^], and development of SAXS data repositories (SASBDB [11^•^], BIOISIS [www.bioisis.net]) show that SAXS has become an invaluable component of the structural biologist’s toolbox.

SAXS is now a robust method for enabling molecular cell biology, providing insight not only into biomolecular shape, but also biomolecular pathway interactions and assembly states, conformational populations within macromolecular ensembles, dynamics of disordered systems, and the evolution of biophysical properties under changing environmental conditions. SAXS remains one of the few structural techniques that can probe macromolecular architecture and dynamics without size limitation under native solution conditions. It furthermore provides multiparameter output on sample quality, particle dimensions and density, and conformational flexibility from a single experiment [7^••^,^12^,13^•^]. Although traditionally considered a low-resolution technique, high-resolution differences in macromolecular conformations can be reliably detected by quantitative comparison of X-ray scattering profiles or SAXS-constrained modeling [14^••^, 15^••^, 16]. When combined with high-throughput (HT) sample acquisition, as pioneered by Hura *et al.* [12], the ability to detect and translate conformational trajectories into functional outcomes across multiple size ranges has greatly extended applications of biological SAXS beyond simple shape characterization. Looking ahead, SAXS is emerging as a method to examine the nanoscale of large cellular machineries and their coordinated interactions. Moreover, SAXS is increasingly able to bridge from the nanoscale into the mesoscale of supramolecular interactions, cellular infrastructure, and interactomes, where electrostatic, mechanical, thermal, and bonding energies of macromolecules share similar orders of magnitude [17]. Thus, SAXS is a uniquely versatile and practical HT method, providing a complete, resolution-limited measure of ordered and disordered molecular states, spanning individual protein folds to the subcellular mesoscale.

Here, we present advanced applications of SAXS, which interrogate biophysical properties and states of macromolecules, as well as their structures, allowing functional insight. We first survey recent advances in SAXS data collection and analysis, building upon the SAXS review by Rambo and Tainer [18] and our earlier work defining pathways from crystal structure snapshots [19]. From there, we examine how SAXS can characterize macro-molecular flexibility and conformational ensembles, uncover biophysical landscapes, and enable applications in HT screening, extending from ligand and co-factor binding to frontiers in drug discovery. We conclude by considering SAXS in the emergent integrative era of structural and molecular biology, where multiple and increasingly sizeable data sets are coming to bear on complex subcellular structures and where the available structural landscape itself is expanding with the rise of genomic information [20].

## SAXS essentials - one experiment, many measurements

**Figure 1:**
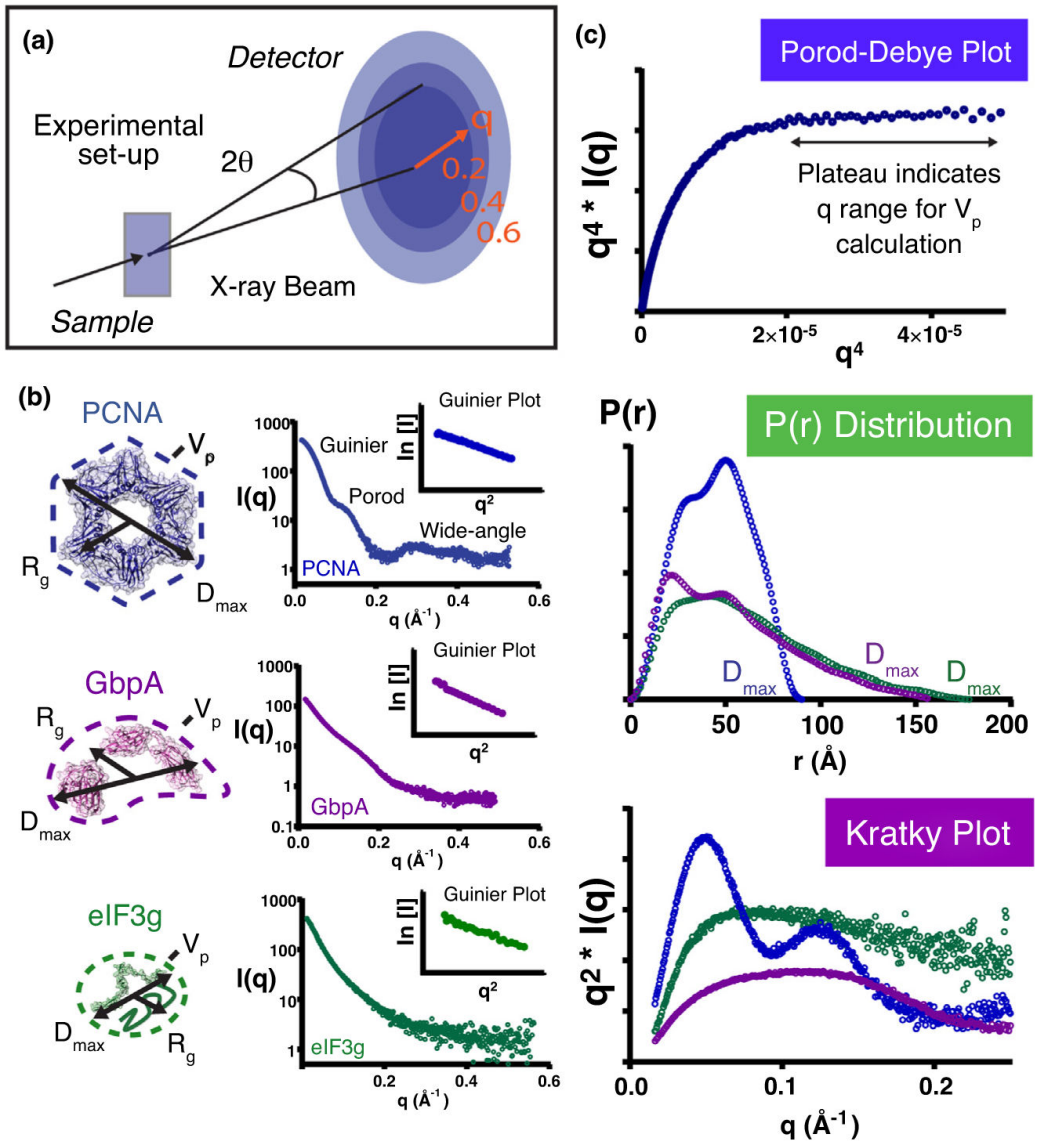
SAXS essentials-one experiment, many measurements. **(a)** A single scattering experiment can provide multiple measures of macromolecular structure. In the basic SAXS experiment, macromolecular solutions are exposed to an X-ray beam, and scattered X-rays are recorded on a detector. Azimuthal integration of the recorded intensity at each q-value, subsequent subtraction of buffer scattering, and extrapolation to infinite dilution (to minimize effects of interparticle interference) yields the one-dimensional X-ray scattering profile, *I*(*q*), that is used to probe molecular geometry and dynamics, **(b)** SAXS profiles are displayed for well-folded, oligomeric PCNA (purple, PDB: 1AXC, SASDBD7), modular GbpA (pink, PDB: 2XWX, SASDAA4), and disordered elF3g (green, PDB:4U1E). The scattering profiles of well-folded macromolecules exhibit elevations and dips (PCNA), while unfolded systems exhibit ‘flat,’ featureless scattering curves (elF3g). The scattering profile of GbpA, which contains ordered domains connected by flexible linkers, exhibits features that are smoothed. Linear transformation of the Guinier region of *I*(*q*) (inset plots) provides an estimate of the radius-of-gyration (*R*_*g*_). **(c)** Mathematical transformations of experimental *I*(*q*) profiles yield additional structural information. The Porod-Debye transform is used to identify the scattering profile’s Porod region for calculating the Porod volume (*V*_p_) of well-folded macromolecules. Fourier transformation of *I*(*q*) yields the real-space, paired-distance distribution, *P*(*r*), with maximum dimension, *D*_max_. The shape of the Kratky transform provides a qualitative assessment of the degree of macromolecular folding or compactness. Well-folded macromolecules exhibit parabolic Kratky curves, which converge toward the baseline at high-*q* values (PCNA), while unfolded systems exhibit hyperbolic Kratky transforms (elF3g). The non-parabolic profile of modular GbpA reflects the flexibility of its linked domains.

In its most basic form, the biological SAXS experiment captures the pattern of X-rays scattered from the electrons that compose a macromolecular solution. The important angular range for shape information on biological macromolecules typically lies between 0.03° and 5° and is best captured by placing a detector 1.5 m or more away from the sample. The particle scattering intensity, *I*(*q*), is a function of all inter-atomic (electron-pair) distances contained within a macromolecule:

(1) $$I(q)=4\pi \int_{0}^{D_{max}}P(r)\frac{\text{sin}(qr)}{qr}dr$$

where *r* is the distance between electron pairs within the macromolecule and *D*_max_ is the maximum of these distances [7^••^] (Figure 1). Scattering intensity is a function of the momentum transfer, *q*:

(2) $$q=\frac{4\pi \text{ sin}(\theta )}{\lambda }$$

where *2θ* is the scattering angle relative to the path of the X-ray beam, and λ is the X-ray wavelength (Figure 1). Importantly, the momentum transfer *q*, reported in Å^−1^ (UK/US) or nm^−1^ (EU), defines the scattering curve in reciprocal space independent of detector distance and wavelength (λ).

Once a measured scattering curve has been corrected for buffer scattering, mathematical transformations of *I*(*q*) (implemented in standard SAXS analysis packages [21^••^,^22^], https://bll231.als.lbl.gov/scatter/) yield information on molecular geometry and sample integrity.

Key examples of these analyses include the Guinier approximation of the low-q region of the scattering curve to estimate the radius-of-gyration (*R*_g_), assessment of the Porod volume (*V*_p_) of the molecular scattering envelope, Fourier transformation of *I*(*q*) to yield the real-space pair-distance distribution of the macromolecule, *P*(*r*), and Kratky transformation, which provides a qualitative assessment of the compactness or flexibility of the scattering particle [7^••^, 16] (Figure 1b, c). Guinier analysis of the low-q scattering signal can also detect sample aggregation and radiation damage, reflected as non-linearity within the Guinier transform and a rise in *R*_*g*_ and *I*(*0*) (the extrapolated zero-angle scattering intensity) with increasing exposure time.

## Moving toward higher signal and experimental throughput

While scattering experiment essentials have not changed, advances in measurement speed and sensitivity are proving to be game changers. In tandem with the detector revolutions in X-ray crystallography and electron cryomi-croscopy (cryoEM), direct photon detectors at SAXS beamlines have improved detection of weak scattering signals from dilute and limited samples while reducing exposure and consequent radiation damage [2,23,24]. A lack of detector dark current and lowered readout noise improves baseline stability and reduces recorded noise within the scattering curve, enabling sample concentrations of 0.5–1.0 mg/mL. The direct detection of X-ray photons, combined with advances in detector readout technology, permits readout rates within the millisecond regime. Increased data collection speed allows shorter, more frequent exposures of SAXS samples, mitigating radiation damage effects and allowing users to utilize early damage-free frames for merging and analysis (sibyls.als.lbl.gov/ran). With the new detectors, virtually every SAXS experiment can essentially become a time-resolved experiment at synchrotron beamlines, and sample solutions can be directly monitored as they emerge from size-exclusion chromatography.

Improvements in sensitivity and readout provided by direct detectors and innovations in capillary sample flow cells have spurred the rapid rise of size-exclusion chromatography coupled (SEG)-SAXS [25^•^,^26^] and time-resolved (TR)-SAXS [27^•^,^28^,^29^]. The advent of SEG-SAXS allows spatial separation according to size, whereas continued improvements in TR-SAXS enable temporal separation of changes in conformation and assemblies. SEG-SAXS applications have proved particularly powerful in isolating monodisperse species from polydisperse or aggregating samples, thereby yielding structural information on transient macromolecular conformations and complexes inaccessible by static observation (*infra vide*). Moreover, combining SEG-SAXS with singular value decomposition (SVD) methods, such as Evolving Factor Analysis (EFA) [30^•^], can yield unique scattering profiles from co-eluting species. The increased ability to automate buffer equilibration and sample loading is guiding SEG-SAXS toward the high-throughput regime.

TR-SAXS experiments capture transient and evolving macromolecular conformations occurring on timescales of microseconds to days. The exact time resolution depends upon the trigger initiating macromolecular changes, whether laser irradiation (light), pressure or temperature jumps, or most commonly, microfluidic mixing with continuous or stopped-flow devices [27^•^]. TR-SAXS coupled with rapid mixing can monitor biomolecular transitions occurring on timescales of microseconds to milliseconds. While sample consumption remains high for TR-SAXS, a single experiment can capture multiple states along a conformational trajectory, yielding critical kinetic insights into biological processes.

Conventional SAXS has become a true HT structural technique with advances in automated sample handling, sample cell design, and sample preparation. Synchrotron SAXS beamlines have now demonstrated acquisition rates of 30–60 min per 96-well plate (http://bll231.als.lbl.gov/) [2,31], allowing screening experiments to take place in conjunction with structural characterization. Currently, sample cell washing occupies the highest percentage of a plate’s acquisition time, and parallelized sample loading and washing is expected to lower acquisition rates by half or further. While standard liquid handling robotics can be used to prepare 96-well plates for HT-SAXS, microfluidic sample platforms, such as the LabDisk for SAXS and Photonic Lab-on-a-Chip, are an active area of development to reduce sample volume and preparation time [32,33]. These devices allow rapid multiplexing of buffer and screening conditions concurrently with preparation of sample dilution series, using minimal material (2.5 μL for LabDisk). Continued innovation in microfluidic sample devices is expected to further enable SAXS as a technology for mainstream HT screening.

## HT approaches to SAXS analysis

As SAXS has entered the HT era, approaches for assessing and interpreting large-scale SAXS data sets are critically needed. Data quality evaluation and analysis have traditionally required time-intensive manual processing and assessment. Thus, the emergence of automated, on-line data analysis pipelines to assess, process, and analyze multi-sample data sets are critical [34,35] (https://www-ssrl.slac.stanford.edu/~saxs/analysis/saxspipe.htm). The robustness of high-throughput data assessment has been examined using the *SAXSstats* protocol of Grant *etal*. [34]. The SAXS analysis program Atsas also includes a high-throughput analysis module SAS-FLOW, which can move SAXS data from background subtraction to modeling [35].

**Figure 2:**
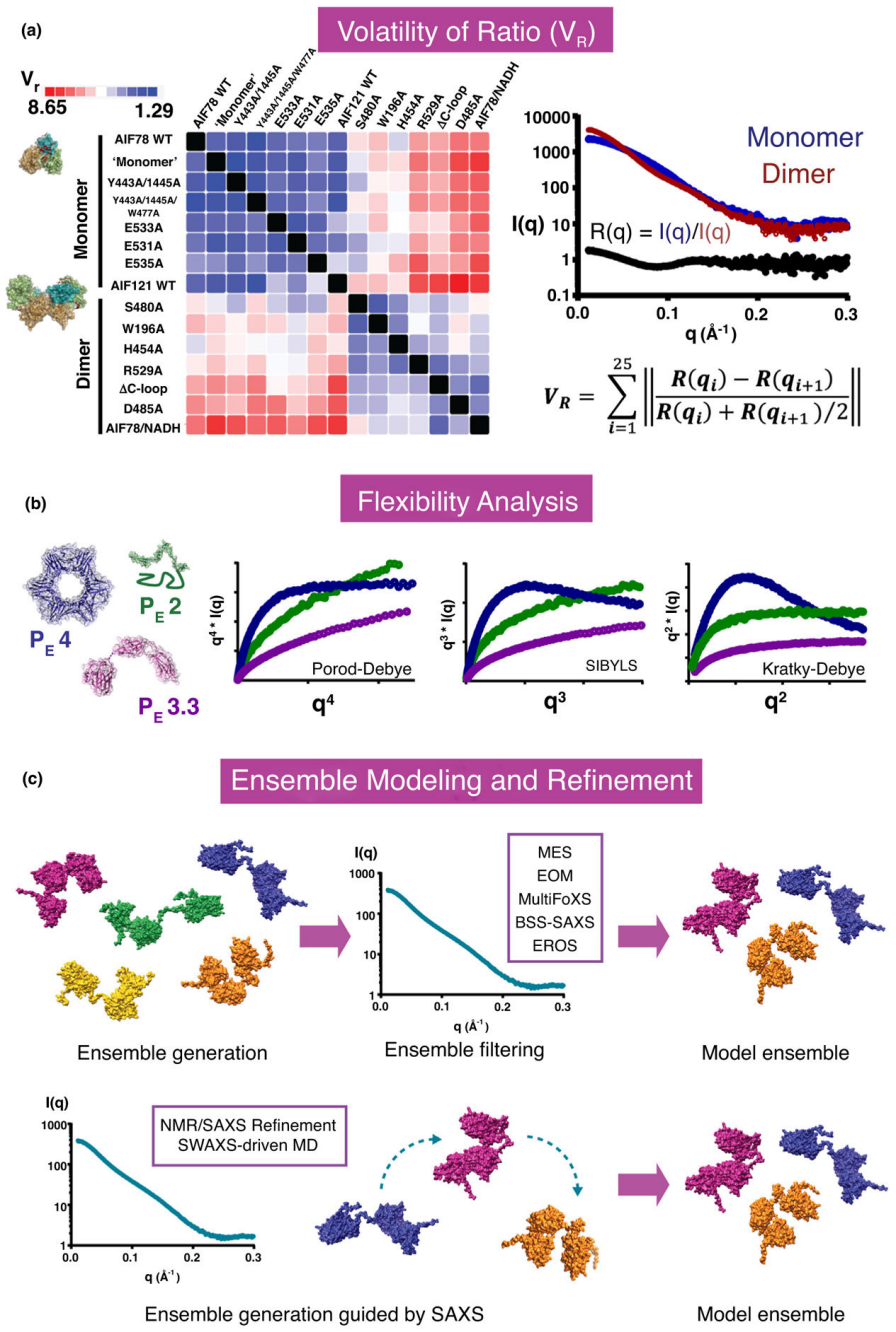
The expanding SAXS analysis toolbox: assessing similarity and biomolecular flexibility. **(a)** Volatility of ratio (*V*_R_). The Volatility-of-Ratio (*V*r) metric quantifies high-resolution conformational differences between paired SAXS curves and importantly provides equal weighting between low-resolution and high-resolution g-space. High similarity follows low *V*_*R*_ values. Assembling *V*_*R*_ values into SAXS Similarity Matrices (SSM) and applying clustering routines reveals conformational populations, as shown for a library of mutants mimicking monomeric (blue) or dimeric (red) AIF (adapted with permission from Ref. [36]). **(b)** Flexibility Analysis. The Porod exponent (*P*_E_) quantifies a power-law relationship describing the degree of foldedness versus flexibility in a sample. Complementary power transforms of the scattering curve by *q*^*2*^, *q*^*3*^, and *q*^*4*^ enable detection of biomolecular flexibility. The well-defined PCNA architecture yields the maximum Porod exponent of 4 for a folded particle, reflected in the plateau of its Porod-Debye plot [*q*^*4*^·*I*(*q*)] (purple trace). In contrast, disordered elF3g exhibits the minimum Porod exponent of 2 for a flexible Gaussian coil, reflected in the plateau of its Kratky-Debye plot [*q*^*2*^·*I*(*q*)] (green trace). Flexible, modular GpbA exhibits an intermediate Porod exponent of 3.3, reflecting its mixture of ordered domains and flexible linkers. For GbpA, plateaus are observed in all three power transforms at different *q*-ranges; for the *q*-range displayed here, plateaus are observed in the Kratky-Debye plot [*q*^2^·*I*(*q*)] (pink trace). Plateau formation within the SIBYLS plots is particularly diagnostic for biomolecules that contain both ordered and disordered elements, **(c)** Ensemble Modeling and Refinement. Modeling conformational ensembles from SAXS data has traditionally been accomplished by screening conformers generated by simulation algorithms against the scattering profile, *l*(*q*), to identify a grouping with the best fit to the data. SAXS-guided molecular dynamics (MD) simulations and hybrid NMR/SAXS refinement algorithms more robustly sample conformational space relevant to the ensemble by incorporating energy terms referencing the *I*(*q*) scattering profile. Exemplary DNA Ligase III models and SAXS data were prepared with MultiFoXS [53,179] (PDB: 3L2P).

For distinguishing *I*(*q*) differences in a screening context, rapid and robust methods are key for comparing and detecting differences among a population of scattering profiles. The volatility-of-ratio (*V*_R_) parameter developed by Hura *et al.* [14^••^] assesses differences for the normalized, binned ratio of two scattering curves, *R*(*q*), where *R*(*q*) *= I*(*q*)_1_/*I*(*q*)_2_ This provides a robust metric for pairwise comparison of scattering curves across the entire resolution range of scattering vectors to define structural similarity objectively (Figure 2). Although valuable, classic pairwise difference metrics, such as *χ*2 and the Pearson correlation coefficient, give increased weight to low-resolution regions of *I*(*q*). In contrast, ratiometric *V*_*R*_ offers even weighting of the entire *q*-range and is thus sensitive to differences at both high and low *q*-values, more effectively detecting sample differences on multiple distance scales. Having calculated *V*_R_ for a population of scattering curves, the resulting *V*_R_ values can be efficiently assembled, clustered, and assessed for trends using a SAXS similarity matrix (https://bll231.als.lbl.gov/saxs_similarity/). This HT, population-level approach to SAXS analysis is robust and objective for a wide range of biological problems, from ligand-induced allosteric states [36] to DNA repair enzyme conformations[14^••^].

## Expanding the SAXS analysis and modeling toolbox

As SAXS experimental set-ups have continued to evolve and develop, SAXS theory and analytical approaches have made similar advances, particularly for the characterization of flexibility and dynamics in biomolecular systems. Although some information on flexibility may be obtained in X-ray crystallography from temperature factors corrected for crystal contacts [37], SAXS directly measures flexibility in solution. Detecting flexibility not only provides insight into molecular architecture and structural changes, but also guides the choice of rigid-body or population-based ensemble approaches when generating molecular models with pre-existing high-resolution structures. Flexibility analysis is also critical for determining whether classical *ah initio* shape reconstruction, implemented by programs such as DAMMIN [38] and GASBOR [39], is appropriate for a system.

The development of the Porod-Debye interpretation of flexibility with Kratky-Debye [*q*^2^·*I*(*q*)], SIBYLS [*q*^3^·*I*(*q*)], and Porod-Debye [*q*^4^·(Iq)] plots and their corresponding quantitation by the Porod exponent (*P*_E_) have enabled objective, quantitative assessment of molecular flexibility and compactness [40^••^,·^41^] (Figures lc, 2b). The presence or absence of a plateau in these three power transforms of the scattering curve *I*(*q*) are assessed to diagnose flexibility. Rigid, well-defined macromolecules exhibit defined plateaus in the Porod-Debye transform [*q*^4^·(Iq)]. Intrinsically disordered systems exhibit plateau formation in the Kratky-Debye plot [*q*^2^·*I*(*q*)]. The SYBILS plot [*q*^*3*^·*I*(*q*)] presents a plateau for systems containing a mixture of rigid and flexible elements, such as flexible, multi-domain proteins. The Porod exponent provides a quantitative measure of the qualitative behavior observed in flexibility plots, assuming values of 2–4 for fully flexible to fully compact systems, respectively.

The recently defined volume-of-correlation (*V*_c_) parameter is the first SAXS invariant to be discovered since the Porod invariant sixty years ago [42^••^]. It is calculated as a scaled ratio of particle volume (*V*_p_) and self-correlation length (*l*_c_) and provides complementary monitoring of changes in molecular conformation for flexible systems [42^••^]. When comparing two matched scattering profiles (i.e. receptor with and without ligand), increases in *V*_c_ are reflective of decreased compactness and increased flexibility, and vice-versa. Pairing *V*_c_ with the radius-of-gyration to form the power-law parameter $Q_{\text{R}}\left(V_{\text{c}}^{2}/R_{\text{g}}\right)$ critically enables direct determinations of hydrated molecular mass of compact and flexible SAXS samples without the need for absolute scaling calibrations [42^••^]. Such concentration-independent methods to assess biomolecular mass are invaluable for discriminating among scattering changes arising from sample assembly formation versus conformational rearrangement [42^••^,^43^,44^•^].

The presence of conformational flexibility in a SAXS sample should steer modeling efforts toward ensemble approaches for flexible systems, when pre-existing high-resolution structures are available (reviewed in the next section). When high-resolution structures are unavailable and flexibility analysis indicates structured macromolecular flexibility, a recently developed *ab initio* shape reconstruction program, DENSS, may provide low-resolution insight into macromolecular architecture. Traditional *ab initio* shape reconstruction programs, such as DAMMIN and GASBOR [39,45,46], optimize placement of spherical beads within a fixed volume restrained by *D*_max_ relative to the *I*(*q*)~derived *P*(*r*) distance distribution, creating a low-resolution shape envelope reflecting macromolecular architecture. Modeling of flexible biomolecules by these *ab initio* methods often fails, however, from penalty restraints requiring a compact model and uniform density.

DENSS (DENsity from Solution Scattering) applies iterative structure factor retrieval directly to experimental scattering data to produce low-resolution electron density volumes [47^•^]. Its advantage over current *ab initio* shape reconstruction algorithms lies in capturing non-uniform biomolecular volumes (e.g. particle cavities) and detecting differences in electron density among different biomolecular phases (e.g. protein versus lipid). Because it allows for non-uniform electron density, DENSS may improve modeling of flexible and disordered systems. A key need for all *ab initio* reconstruction algorithms is full utilization of *I*(*q*) information from the high-q region (q > 0.2 Å). As *I*(*q*) spans two orders of magnitude (10^2^) across *q* space (Figure 1), noise has the greatest impacts on low signal in the high-*q* region. Consequently, low angle *I*(*q*) with high intensity and low noise dominates *ab initio* reconstructions, leaving lower intensity, noisier, high-*q* data underutilized. As the high-*q* signal is being measured with increasing accuracy, this higher resolution data could extend the detail and resolution of *ab initio* models.

Continued developments in *ab initio* modeling have also examined questions of uniqueness and resolution for shape reconstructions. The *ATSAS* analysis module AMBIMETER provides a new aid to assess shape ambiguity before the calculation of the shape reconstruction by determining the uniqueness of the experimental scattering profile relative to a library of shape skeletons [48^•^]. SAXS data exhibiting unique topological shape information are more likely to produce unambiguous *ab initio* modeling results. SASREF, also from *ATSAS*’, utilizes the average Fourier shell correlation (FCS) function across a set of *ab initio* envelope solutions to generate an estimate of envelope resolution and thus a quantitative benchmark for comparing envelope reconstructions from different SAXS curves [49^•^].

## Structural dynamics: capturing functional biomolecular flexibility

SAXS can access both well-defined macromolecular architecture and flexible dynamics simultaneously, revealing functional conformations and dynamics often invisible to static approaches, such as X-ray crystallography and cryoEM. It also captures the complete architecture of the biologically relevant solution ensemble, in contrast to other site-specific solution techniques (NMR, FRET, EPR), which may selectively report from very specific regions of a biomolecule. Multiple approaches are available to model dynamic conformational ensembles encoded in the scattering curve. Current ensemble modeling programs include EOM (Ensemble Optimization Method) [50,51], Minimal Ensemble Search (MES) partnered with BILBOMD [52] or MultiFoXS (Multi Fast X-ray Scattering) [53,54], EROS (Ensemble Refinement of SAXS) [55], and BSS-SAXS (Basis-Set Supported SAXS) [56] (Figure 2c). These programs utilize different approaches to generate starting ensembles for refinement against SAXS data. These include high-temperature, implicit-solvent molecular dynamics on domain linkers (BilboMD), knowledge-based sampling (EOM, MultiFoXS), and coarse-grain molecular dynamics (BSS-SAXS, EROS). Each program has unique advantages to modeling different kinds of biomolecular ensembles. EOM shows success in modeling biomolecular systems with highly fluctuating structures, such as intrinsically disordered proteins (IDPs) [57,58] and RNAs [59]. BILBOMD-MES is optimized for flexibly linked, multi-domain systems or rigid domains with flexible loops or termini [60,61], as are EROS [62,63] and BSS-SAXS [56]. MultiFoXS and its MES approach are targeted toward modeling conformational heterogeneity within well-defined molecules, such as immunoglobulin chains [64,65^•^].

Ensemble modeling approaches have proved critical to revealing the properties of dynamic functional states. Recent scattering studies have probed functional conformations in cytoskeletal actin-binding protein adseverin [66], assemblies from the mammalian circadian clock [67], intrinsically disordered proteins tau and a-synuclein [68,69^••^], multi-domain bacterial carboxylic acid reductase [70] and outer-membrane protein (OMP) chaperone Skp [71], ubiquitin-modified and SUMO-modified PCNA [72,73], dynamic complexes of the non-homologous end-joining (NHEJ) DNA double-strand break repair pathway [57,74,75^••^] DNA conformations in DNA mismatch repair [76], and nucleosome unwrapping [77^••^,^78^].

Moreover, ensemble approaches are significantly extended in their application when combined with SEG-SAXS and TR-SAXS experiments. Combined application of ensemble modeling and SEC-SAXS was essential to characterizing the solution architectures of Ku/ DNA-PKcs/APLF and Ku/XRCC4/DNA Ligase IV/APLF assemblies, which orchestrate NHEJ repair [75^••^]. Studying intact complexes bound to DNA substrate with SEC-SAXS permitted the detection and isolation of scattering signal from stably associated, nonaggregating complexes. Subsequent modeling of the complex was aided by ensemble modeling of intrinsically disordered APLF and the flexibly attached C-terminal domain of Ku80 using BILBOMD-MES. Besides capturing ‘instantaneous’ molecular ensembles, ensemble modeling is increasingly used to monitor evolution of ensembles over time via TR-SAXS. The elegant exploration of nucleosome unwrapping by Chen and colleagues used such ensemble methods to deconvolute DNA conformational changes over progressing SAXS snapshots and to construct kinetic pathways for nucleosome disassembly [77^••^, 78]. Their study also cleverly capitalized upon sucrose contrast-matching of the sample buffer to minimize the scattering signal from protein histones and to maximize the DNA scattering signal for analysis. Similarly, Plumridge *et al.* tracked the progression of magnesium-induced conformational collapse for the tP5abc three-helix junction RNA with TR-SAXS and ensemble fitting from molecular dynamics snapshots [79^••^].

While ensemble methods provide realistic representations of solution conformations, their ability to describe ensembles is often constrained by limitations in fully sampling the available conformational space for subsequent screening against SAXS data. Coarse-grained (CG) and all-atom (AA) molecular dynamics simulations, computed with implicit or explicit solvation, are being used with rising frequency to increase conformational sampling and to aid the interpretation of scattering data [62,73,80–83]. With their reduced particle number and degrees-of-freedom, coarse-grained approaches enable broad and rapid conformational sampling of collective macromolecular motions with a streamlined computational load [84]. At the same time, recent advances in parallelization with GPU (graphics processor unit) technology have made the extended periods of AA simulations (sub-microseconds and longer) accessible to desktop computers. Notably, application of sampling enrichment strategies (accelerated MD, amplified collective motions) are also improving conformational pools for SAXS-driven ensemble selection [85,86].

An innovation in ensemble modeling driven by both GG and AA MD simulations applies the experimental SAXS curve as an energetic restraint in structure sampling and refinement, rather than a comparative reference or a postsampling filter for conformational selection [15^••^,^83^,^87^,^88^] (Figure 2). Hybrid refinement methods, such as those that combine NMR and SAXS data [89,90^•^,^91^], use a similar approach by incorporating a SAXS-fitting term into existing NMR-parameter driven scoring functions. Chen and Hub, however, present a direct refinement method with small-angle and wide-angle scattering data (SAXS/WAXS), using explicit-solvent molecular dynamics (MD) simulations to evolve crystallographic starting models (SWAXS-driven MD) [15^••^]. Their SAXS-guided sampling ensures adequate exploration of the relevant conformational space, while their application of explicit solvent avoids inaccuracies from fitting of the solvent layer and excluded volume, thereby achieving better modeling of higher-resolution wide-angle scattering data. The use of molecular dynamics to model a more accurate solvent layer is also employed by *WAXSiS* [92^•^], which computes theoretical scattering curves from fixed atomic PDB coordinates.

As use of SAXS-guided structural refinement and explicit modeling of macromolecular hydration becomes mainstream, testing how higher-resolution data from wide-angle scattering experiments impacts and improves knowledge of structures and conformational dynamics will be valuable, especially for parsing high-resolution scattering contributions from atomic thermal motions [93]. Conversely, SAXS-guided insights from biomolecular and solvent dynamics may aid in bridging the ‘R-factor gap’ for correlating crystal structures with X-ray diffraction data [94]. Hybrid refinement methods, which utilize multiple sources of structural information (X-ray crystallography, NMR, SAXS, cryoEM) are also poised to benefit from advances in SAXS-based modeling and refinement strategies.

## Probing biophysical landscapes

**Figure 3:**
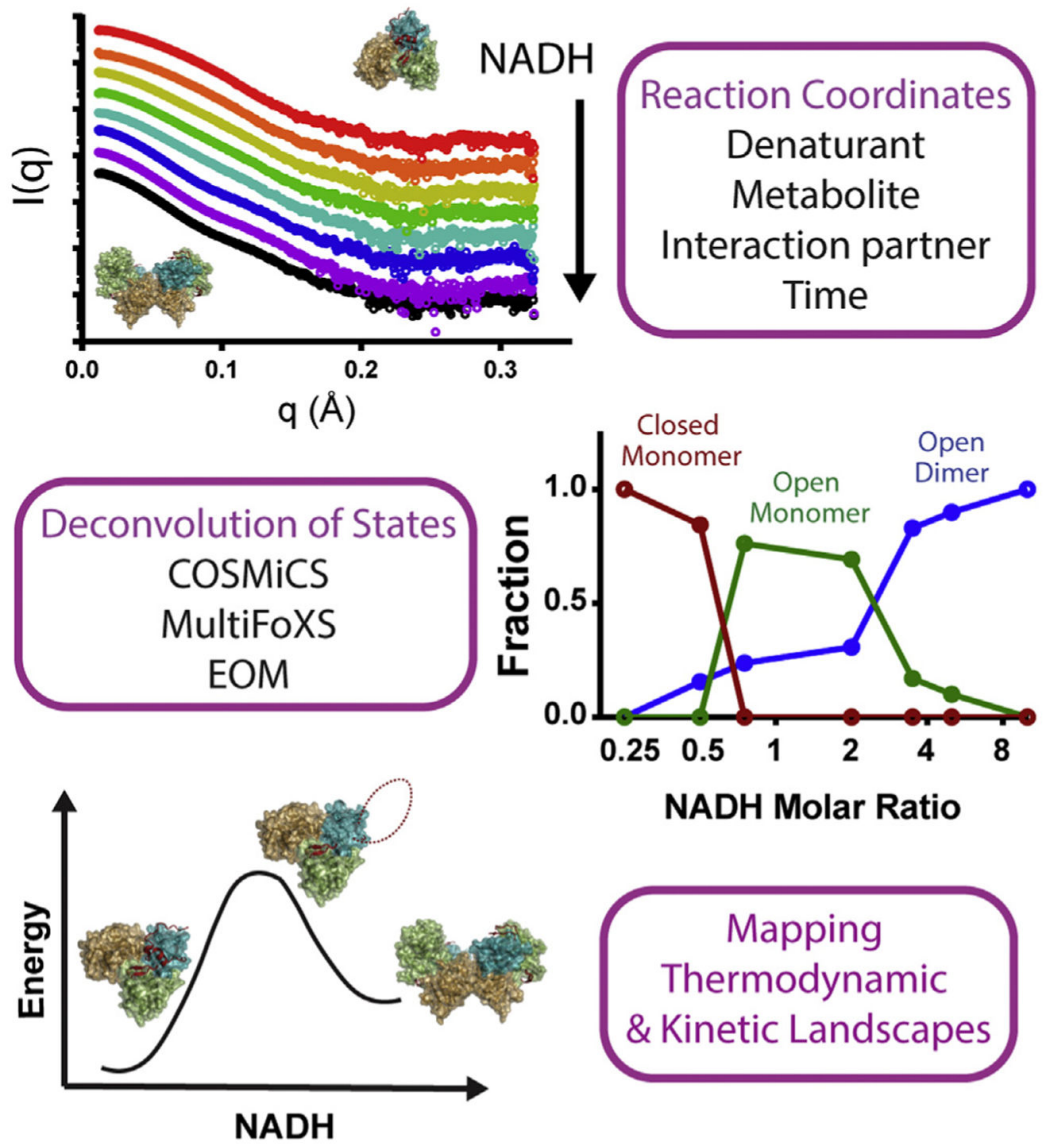
Illuminating biomolecular pathways and energy landscapes with SAXS. The advent of multi-state modeling algorithms for deconvolution has enabled solution architectures to be transformed into reaction coordinates and energy landscapes. Sequential SAXS acquisition on macromolecules under evolving conditions of time, denaturant, metabolites, or binding partners can be analyzed for shifts in conformational populations, using known reference states (FoXS, EOM) or coordinate endpoints (COSMiCS). These evolving ensembles can subsequently be used to derive thermodynamic and kinetic insights on pathway progression. Here, SAXS monitors mitochondrial import and death factor protein AIF as it transitions from monomer to dimeric states upon binding NADH. Multi-state fitting with MultiFoXs identifies three populations: AIF monomer, AIF monomer with an internal 50-residue loop (C-loop) exposed to solvent, and AIF dimer with exposed C-loops.

Beyond establishing functional dynamic structures, SAXS is now a key technology for investigating functional biophysical properties. Biomolecular shape and flexibility encode thermodynamic information, reflective of their folded, multi-conformer, or disordered states, and can be monitored for state changes (Figure 3). SAXS *R*_*g*_ and *P(r)* measurements are increasingly used for proteins [95–100,101^•^] and RNA [41,102–108] to construct temperature and ion-dependent phase diagrams and reaction coordinates for folding. Protein energy landscapes can be assimilated from or validated by SAXS data [109,110]. TR-SAXS accesses biomolecular reaction and pathway intermediates, as exemplified by studies of virus capsid maturation [111], the photocycle of photoactive yellow protein (PYP) [29], and nucleosome disassembly [77^••^], allowing extraction of kinetic information and delineation of conformational trajectories. Notably, the ability to detect and quantify populations of individual species and their complexes within scattering data can reveal thermodynamic interactions among binding partners.

A unique application of multi-species population modeling was reported by Gordeiro *et al.* and provided an analysis framework for using SAXS titration series to monitor and model transient, multi-species interactions, in this case, DNA damage response factor PGNA and its disordered regulatory binding partner pl5^PAF^ [112^••^]. Their study used explicit-solvent MD simulations to model the free binding partners and three potential interaction stoichiometries, from which ensemble-averaged SAXS curves were generated. By use of the ensemble-averaged SAXS curves as a basis set, they globally fitted the experimental scattering data collected across a pl5^PAF^/PCNA titration series to deconvolute fractional binding populations and estimate the *K*_d_. Their approach simultaneously quantified concentration-dependent population distributions of pl5^PAF^/PGNA complexes, while illuminating the heterogeneous architecture of each complex.

Mapping dynamic landscapes of protein-DNA complexes by SAXS has benefited from selective labeling with heavy elements that scatter more strongly than protein or DNA. The average electron density of gold nanocrystals is ~4.6 electrons/Å^3^ compared to 0.44 electrons/Å^3^ for protein or 0.55 electrons/Å^3^ for DNA. For biological SAXS experiments, the scattering signal is scaled by the square of the electron density difference between the scattering object and water (0.33 electrons/Å^3^). Thus, the zero-angle scattering intensity for a gold particle is 1650-fold greater than a protein of equivalent size. With mathematical treatments of gold nanocluster scattering in place [76], their > 1000-fold increased scattering offers powerful opportunities to examine specific distances in complex mixtures. For example, Hura *et al.* successfully used gold-labeled DNA substrates to probe conformational changes on the DNA induced by the *E. coli* mismatch repair factors MutS and MutL. These experiments enabled them to propose a mechanism for base-mismatch recognition, in which the substrate DNA is initially distorted, and then straightened as repair complexes migrate on the DNA.

SAXS can also robustly detect, deconvolute, and quantify kinetic progression of macromolecular aggregation and assembly processes, often associated with significant human diseases. Destabilizing hotspot patient mutations in glycine 93 of Gu, Zn superoxide dismutase (SOD) result in amyotrophic lateral sclerosis (ALS). However, the SOD mutant crystal structures were very similar to the wild-type protein. Nevertheless, SAXS revealed an increased propensity of the mutant enzyme toward aggregated filament formation in solution, corresponding with the clinical severity of ALS [113]. In a similar manner, time-resolved SAXS coupled with a novel data deconvolution approach, COSMiCS (Complex Objective Structural Analysis of Multi-Component Systems), probed amyloid formation by insulin and the E46K α-synuclein Parkinson’s disease mutant [69^••^]. In this application, COSMiCS was used to extract component scattering profiles for an evolving mixture of species (monomer, oligomer, fibril). Experimental *I(q)* scattering profiles collected during the aggregation process and combinations of their mathematical transforms (Holtzer *q·I*(*q*); Kratky *q*^2^*·I*(*q*); Porod *q*^4^*·I*(*q*)) were used as inputs. The inclusion of the *I(q)* transforms, which emphasize different distance scales encoded in the experimental *I(q*) curves, proved important for isolating scattering curves from species along the aggregation trajectory and for estimating their relative populations. Continued innovation in TR-SAXS, as well as SEC-SAXS, will enable further exploration and development of biophysical applications for insight into fundamental biochemical processes and human pathologies.

## HT screening with SAXS: current and emerging applications

HT data collection platforms have spurred the expansion of screening applications using SAXS [114]. Current among these are rapid validation of protein engineering design targets [101^•^,^115^–^118^], micro-screening of macromolecular crystallization conditions [119,120], characterization of protein mutant/variant libraries [36,113,121–123], profiling ligand/metabolite binding [14^••^], assaying for protein-RNAand protein-ligand interactions [14^••^, 124], and assessing antibody formulations [125–127]. SAXS offers the dual benefit of facilitating screening endpoints in solution, while providing multi-parameter architectural read-outs on each system.

SAXS has proved increasingly significant for synthetic biology, facilitating efficient design and optimization of nanoscale biological materials. For example, SAXS was used to screen self-assembling cyclic homo-oligomers and to link nanoscale architecture with rational design of protein interfaces [117]. In a similar manner, SAXS determined conformational classifications of self-assembling protein cages and interrogated cage stability under a range of solvent, pH and salt conditions [101^•^]. Notably, these authors created a custom, theoretical conformational landscape for benchmarking their cage designs with SAXS. Conformational snapshots were generated by a Chimera morph between compact and symmetrically open cage structures. The authors then simulated SAXS curves for these conformational snapshots and used this conformational benchmark to interpret the experimental impact of exposing protein cages to varying solvent conditions. Their analysis made use of simultaneous plotting of theoretical and experimental data in *V*_R_ similarity matrices and force plots, which represented each dataset as a node and scale distance between nodes according to V_R_ similarity (https://bll231.als.lbl.gov/saxs_similarity/). This ability to compare and rapidly assess biomolecular materials against targeted designs positions SAXS to play a key role in the design cycle of nanoscale bioengineering.

In the same way, HT-SAXS assessments have and will continue to provide feedback on macromolecular targets traversing protein biochemistry and crystallography pipelines. Success in protein crystallography relies first upon effective construct design, and SAXS provides a ready means for determining and selecting stable protein constructs from prepared libraries, identifying constructs which minimize aggregation and internal flexibility. SAXS is also well positioned to identify optimum solvents to support protein construct stability once a construct has been selected. The recent demonstration of SAXS’s ability to measure second virial coefficients for varying lysozyme and salt concentrations on a microfluidic chip [119] is further support for the potential of SAXS to aid in identifying conditions favorable for crystallization.

While SAXS has found diverse HT applications, it still remains underutilized in arenas of small-molecule screening and drug discovery. Nevertheless, SAXS excels in detecting ligand impact on macromolecular structure: the formation, perturbation, and disruption of protein complexes; allosteric rearrangement of protein domains; and enhanced or restrained polypeptide flexibility. Examples of physiologic small-molecule ligand interactions accessible by SAXS have included receptor-ligand binding [128], co-factor interactions [36,129], metal ion binding [130], and UV photo sensing [131]. Moreover, ensemble readout from SAXS is well suited to detecting selective stabilization of transient conformations by ligand interactions. Development of allosteric modulators of protein ensembles has come increasingly into focus for drug targeting, as these ligands avoid competitive interplay with endogenous ligands [132–134]. The move to target small-molecules toward protein complexes and assemblies to more effectively modulate signaling pathways is well aligned to these advantages of SAXS-based approaches for screening and structure-function analysis. As the useful resolution range of the scattering curve expands, SAXS may find a place in providing read-out of subtle target-ligand interactions.

## The integrative structural biology era

**Figure 4:**
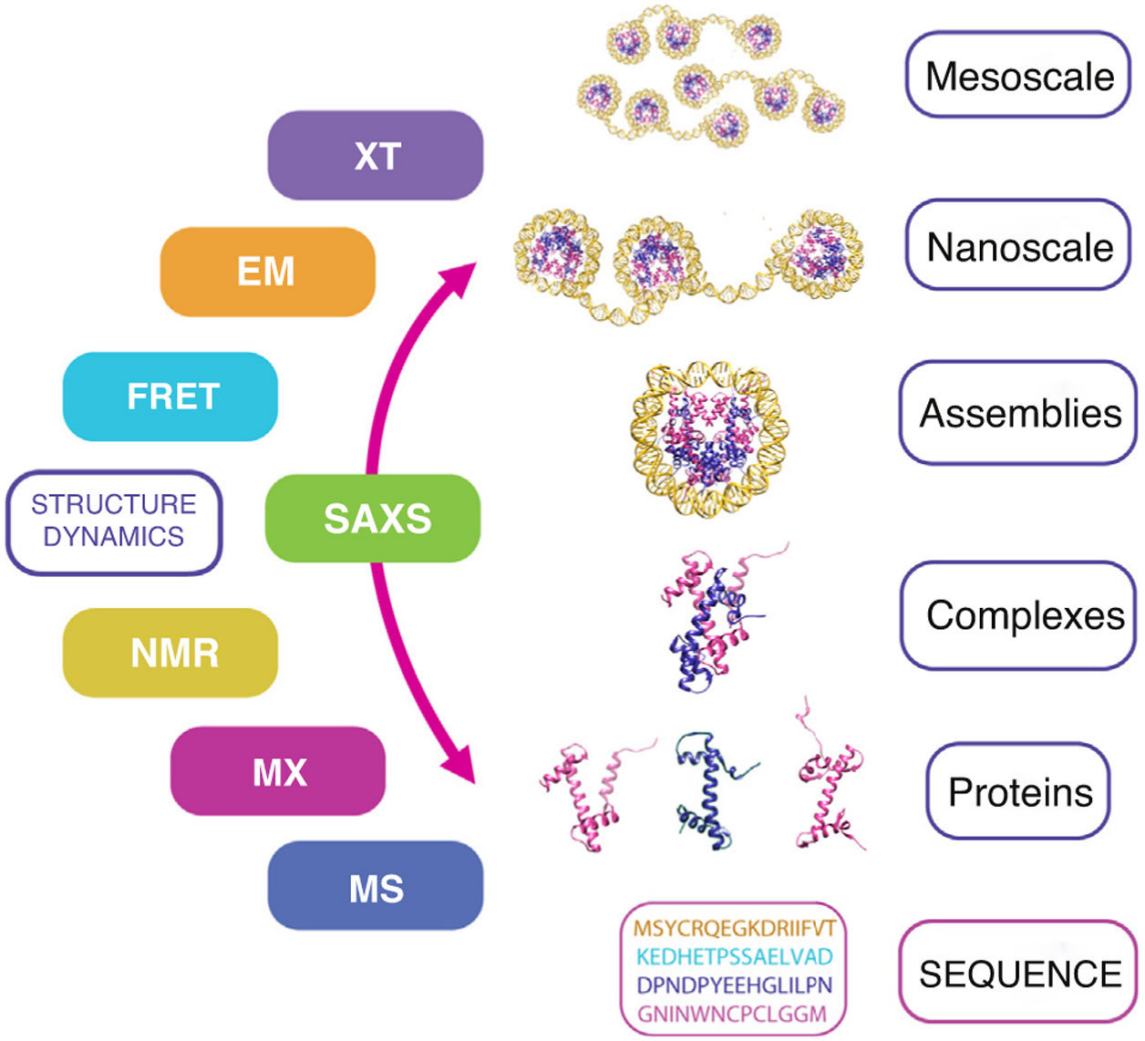
Integrative structural biology: moving from macromolecular assemblies to cellular structures. The era of integrative structural biology brings multiple techniques to bear on multi-scale macromolecular structures, including X-ray tomography (XT), electron microscopy (EM), fluorescent resonance energy transfer (FRET), small-angle X-ray scattering (SAXS), nuclear magnetic resonance spectroscopy (NMR), macromolecular crystallography (MX), and mass spectrometry (MS). Structures of the human nucleosome adapted from PDB: 5AV8.

The twenty-first century has heralded the integrative era of structural biology, where comprehensive descriptions of macromolecular architecture and function are assembled from multiple, complementary structural techniques [135–137]. For many years, SAXS has extended conformational and oligomeric information from atomic resolution crystal structures [138–142], aided NMR-driven structural refinement and model-building [82,89–91,143–149], provided global read-out to complement NMR dynamics measurements [150], enabled visualization of protein complexes from crystallized or computationally modeled components [151,152], and informed *ab initio* protein fold modeling [99^•^]. With improved methods for integrative computational modeling [153,154^••^,155^••^], advances in native and cross-linking mass spectrometry (MS) analysis [156–158], integration with single-molecule methods [159], and the emergence of the cryoEM revolution [160,161], SAXS is primed to provide integrative conformational information for large macromolecular assemblies *in vitro*, as well as biological complexes studied *in situ* by cryo-soft X-ray and cryo-electron tomography [162–164] (Figure 4).

Complementary validation and interpretation of macromolecular assemblies from cryoEM or cryo-tomographic methods using SAXS data are already mainstream [165–168]. Global metrics for evaluating integrative structural models generated from SAXS and complementary data sets, however, remain rare. Multi-data refinement platforms, such as the Integrative Modeling Platform (IMP), have developed tools for synthesizing multiple sources of spatial restraints to drive model-building and refinement [155^••^,^169^,^170^], and efforts by the world wide Protein Data Bank (wwPDB) and others have begun to lay groundwork for the curation and validation of integrative/hybrid structural models [171^•^,^172^]. While efforts by platforms such as IMP have made impressive headway in bringing diverse data sources to bear on hybrid models, a key advance remains to be made in the pursuit and development of confidence-weighted multi-data refinement methods to capitalize upon the common structural information encoded in X-ray crystallography, cryoEM, and SAXS data.

Looking toward the future, structural biology is poised to extend the pursuit of macromolecular assemblies and machinery to nanoscale and mesoscale cellular structures. Notable recent examples have included the impact of Tau variants on microtubule crowding [173], the architecture of nucleosome fibers [174^•^, 175], and bacterial nucleoid compaction [168^•^]. With time-resolved methods, SAXS has the potential to investigate the biochemical determinants of more dynamic supramolecular assemblies, such as phase-driven coalescence of chromatin subcompartments [176], nucleation of stress granules [177], and diffusion recovery of DNA repair foci. These novel phase separations may entail Turing pattern formation and could be examined by SAXS analytics such as *V*_c_, which reports on voids within assemblies [42^••^]. Such dynamic biomolecular condensates represent a frontier for extending SAXS into the study of cellular structures, linking nanoscale and mesoscale in cell biology. In a similar manner, the exponential increase in genomic sequencing data across species and disease states also presents opportunities and challenges for extracting structural information to aid in predicting phenotypic outcomes. Here, SAXS can link important human protein targets to accessible yeast and bacterial model protein systems to inform human molecular biology and disease [178]. Combining such approaches with rapid HT-SAXS analyses can provide opportunities for translating disease-specific and species-specific variations in target sequences into libraries of three-dimensional architectural information, reporting on functional variation that can be leveraged for diagnostic output.

## SAXS: today and future horizons

The past two decades have established biological X-ray scattering as a mainstay of structural biology and expanded the paradigm for interpreting macromolecular function through supramolecular architecture. SAXS is well established in revealing the shape, conformations and assemblies of biological systems. As the field continues to evolve and illuminate complex biological problems, novel applications of HT-SAXS, SEC-SAXS, and TR-SAXS will extend the spatial and temporal resolving power of this technique even further. Biological SAXS has and will continue to capitalize upon computational advances to drive interpretation of scattering data towards higher resolution and further insight into macromolecular shape, assembly states, flexibility, and conformational ensembles. SAXS has also become a powerful tool for tracking biophysical states associated with folding, unfolding, and aggregation and for assaying biochemically relevant ligand interactions. The HT scale of SAXS has facilitated its use in biotechnological applications, such as synthetic biology and protein construct screening, and is well positioned to aid in drug discovery and diagnostic structure-function analyses of disease-causing and cancer-causing mutations. Looking forward to the ‘SAXS revolution’ over the next decade, we anticipate that biological X-ray scattering will continue to be a driver in integrative structural biology, empower investigation of nanoscale/mesoscale cellular structures, and sustain a role in mapping novel and dynamic functional architectures from the global genome.
